# BRASH Syndrome: A Case of Hyperkalemia, Bradycardia, and Renal Dysfunction Triggered by Missed Dialysis.

**DOI:** 10.51894/001c.144201

**Published:** 2025-09-30

**Authors:** Kesava Manikanta Achuta

**Affiliations:** 1 Internal Medicine Garden City Hospital

## 56

### INTRODUCTION

BRASH syndrome is a constellation of bradycardia, renal failure, atrioventricular (AV) nodal blocker use, shock, and hyperkalemia. It is caused by the synergism of AV nodal blockers and renal impairment, increasing the effect of AV nodal blockade.

The most common triggering causes include hypovolemia, uptitration of antihypertensives, digoxin intake, beta blocker and calcium channel blocker toxicity, renal failure and hyperkalemia. Here we present a patient who came with clinical picture of BRASH syndrome, in whom early diagnosis and prompt treatment led to successful outcome.

### CASE PRESENTATION

An 86-year-old female with history significant for end stage renal disease on hemodialysis, hypertension, atrial fibrillation, dementia, hypothyroidism and depression presented with lethargy and being minimally responsive for the past few days. She missed 3 sessions of dialysis. On presentation, temperature was 98.6 F, blood pressure 78/46 mm Hg, heart rate 31/min, respiratory rate 12/min. Physical examination was notable for generalized weakness, bradycardia, bibasilar crackles on auscultation, and bilateral lower extremity edema. Labs showed a hemoglobin of 6.9g/dl, potassium 7.2mmol/L, phosphorus 8.7mg/dl, creatinine 8.59mg/dl, urea 117mg/dl and troponin of 82ng/L. Telemetry showed atrial fibrillation with a HR of 31/ min which improved to 59/ min post intravenous Atropine 1mg. Echo is normal with preserved EF of 60%.

Hyperkalemia was treated with shifters along with emergent hemodialysis and blood transfusion. Follow up labs showed potassium of 4 and creatinine of 4.6 after Hemodialysis. Sepsis and cardiac workup were negative. Eventually, she was discharged with education regarding triggers, ensured compliance with regular dialysis.

### DISCUSSION

With aging population, who often have a lack of renal reserve with baseline renal dysfunction, with lower blood pressure targets for the management of hypertension, BRASH syndrome frequency is increasing. In our case, it started with hyperkalemia, worsening renal perfusion, and decreased clearance of AV blockers, all factors acting synergistically to result in worsening bradycardia leading to hypotension which further impaired renal function leading to a vicious cycle. Prompt identification of BRASH syndrome is essential to prevent unnecessary pacemaker placement due to reversible symptomatic bradycardia and prevents management of individuals components. Hence, more awareness and understanding are needed about the syndrome.

